# Impact of structural biology and the protein data bank on us fda new drug approvals of low molecular weight antineoplastic agents 2019–2023

**DOI:** 10.1038/s41388-024-03077-2

**Published:** 2024-06-17

**Authors:** Stephen K. Burley, Amy Wu-Wu, Shuchismita Dutta, Shridar Ganesan, Steven X. F. Zheng

**Affiliations:** 1 Research Collaboratory for Structural Bioinformatics Protein Data Bank, Institute for Quantitative Biomedicine, Rutgers, The State University of New Jersey, Piscataway, NJ 08854 USA; 2 Rutgers Cancer Institute of New Jersey, Robert Wood Johnson Medical School, New Brunswick, NJ 08903 USA; 3 Research Collaboratory for Structural Bioinformatics Protein Data Bank, San Diego Supercomputer Center, University of California, San Diego, La Jolla, CA 92093 USA; 4 Department of Chemistry and Chemical Biology, Rutgers, The State University of New Jersey, Piscataway, NJ 08854 USA

**Keywords:** Structure-based drug design, Drug development

## Abstract

Open access to three-dimensional atomic-level biostructure information from the Protein Data Bank (PDB) facilitated discovery/development of 100% of the 34 new low molecular weight, protein-targeted, antineoplastic agents approved by the US FDA 2019–2023. Analyses of PDB holdings, the scientific literature, and related documents for each drug-target combination revealed that the impact of structural biologists and public-domain 3D biostructure data was broad and substantial, ranging from understanding target biology (100% of all drug targets), to identifying a given target as likely druggable (100% of all targets), to structure-guided drug discovery (>80% of all new small-molecule drugs, made up of 50% confirmed and >30% probable cases). In addition to aggregate impact assessments, illustrative case studies are presented for six first-in-class small-molecule anti-cancer drugs, including a selective inhibitor of nuclear export targeting Exportin 1 (selinexor, Xpovio), an ATP-competitive CSF-1R receptor tyrosine kinase inhibitor (pexidartinib,Turalia), a non-ATP-competitive inhibitor of the BCR-Abl fusion protein targeting the myristoyl binding pocket within the kinase catalytic domain of Abl (asciminib, Scemblix), a covalently-acting G12C KRAS inhibitor (sotorasib, Lumakras or Lumykras), an EZH2 methyltransferase inhibitor (tazemostat, Tazverik), and an agent targeting the basic-Helix-Loop-Helix transcription factor HIF-2α (belzutifan, Welireg).

## Introduction

X-ray protein crystallography and structure-guided approaches have been mainstays for drug discovery for more than two decades [1, 2]. Atomic-level, three-dimensional (3D) structures of biological macromolecules inform our understanding of target biology (reviewed in [3]), and provide important insights into target druggability for both small-molecule and/or biologic agents (reviewed in [4]). Today, macromolecular crystallography (MX) and 3D electron microscopy (3DEM) are routinely used in most large and many small biopharmaceutical companies for structure-guided optimization of affinity of small-molecule screening hits and lead compounds [1]. 3D biostructure data can also aid in surmounting some of the myriad challenges (e.g., avoiding unwanted off-target binding) inherent in turning biochemically active compounds into potent, drug-like molecules suitable for safety and efficacy testing in animals and humans [5]. Finally, starting points for medicinal chemistry campaigns (i.e., selectively binding chemical scaffolds) can be identified via fragment screening using nuclear magnetic resonance spectroscopy or NMR [6], MX [7], and 3DEM [8].

Public-domain 3D biostructure information generated using MX, 3DEM, or NMR is distributed on an open-access basis by a singular global data resource, known as the Protein Data Bank (PDB [9]). When the PDB was established in 1971 as the first open-access digital data resource in biology, it housed only seven protein structures [9]. Today, the PDB is regarded as a global public good vital to basic and applied research and education/training across the biological and biomedical sciences. In the spring of 2024, the PDB housed >220,000 experimentally determined, atomic-level 3D structures of biological macromolecules (i.e., proteins, DNA, and RNA), many of which have been visualized in the act of binding one or more small-molecule ligands, including United States Food and Drug Administration (US FDA) approved drugs. Since 2003, the PDB has been managed jointly according to the FAIR Principles of Findability-Accessibility-Interoperability-Reusability [10] by the Worldwide Protein Data Bank (wwPDB) partnership [11, 12], including the US Research Collaboratory for Structural Bioinformatics Protein Data Bank or RCSB PDB [13–15], Protein Data Bank in Europe [16], Protein Data Bank Japan [17], Protein Data Bank China [18], Biological Magnetic Resonance Data Bank [19], and the Electron Microscopy Data Bank [20].

The RCSB PDB (RCSB.org) headquartered at Rutgers, The State University of New Jersey (with additional performance sites at the University of California San Diego and the University of California San Francisco) serves as the US wwPDB data center and as the wwPDB-designated Archive Keeper for the PDB. On two previous occasions, we have reviewed the impact of structural biologists and PDB structures on US FDA drug approvals. Initially, we examined 210 new drugs approved by US FDA 2010–2016 across all therapeutic areas [21], and determined that open access to nearly 6000 atomic-level 3D structures of molecular targets stored in the PDB archive facilitated discovery and development of 185 (~88%) of these new medical entities (NMEs). Subsequently, we focused on 79 new antineoplastic agents with known molecular targets approved by US FDA 2010–2018 [22], and determined that open access to PDB data facilitated discovery and development of >90% of these life-changing drugs. More detailed analyses of the 54 low molecular weight (LMW) NMEs for cancer treatment approved by US FDA 2010–2018 revealed that >70% were the product of structure-guided drug discovery (SGDD) efforts at biopharmaceutical companies.

Since these analyses of the impact of PDB data on drug discovery and development were published by RCSB PDB, the world of structural biology underwent a seismic shift with emergence of new software tools that rely on artificial intelligence/machine learning (AI/ML) methods to predict protein structure from amino acid sequence alone at accuracies comparable to lower-resolution experimental structures. Development of AlphaFold2 [23], RosettaFold2 [24], etc. would not have been possible without open access to complete, rigorously validated, expertly biocurated 3D biostructure data archived in the PDB [25]. More recently, AI/ML methods, similarly dependent on open access to PDB data, have been developed to predict how proteins bind small molecules and other proteins (e.g., RoseTTAFold All-Atom [26], AlphaFold3 [27]). These new tools for predicting how small molecules bind to proteins go beyond a plethora of previously developed computational approaches to structure-based drug discovery based on PDB data (e.g., docking methods used in virtual screening and lead optimization, free energy perturbation methods for predicting ligand affinity, and statistical and data-driven tools for analyzing and designing protein ligand complexes) many of which are used across the biopharmaceutical industry.

Herein, we review the ways that structural biologists and open access to PDB data facilitated discovery and development of 34 new antineoplastic LMW-NMEs approved by US FDA 2019–2023. In addition to an aggregate review of PDB impact on new drug approvals, illustrative case studies are presented for six first-in-class small-molecule anti-cancer drugs, including a selective nuclear export Exportin 1 inhibitor (selinexor, Xpovio), an ATP-competitive CSF-1R receptor tyrosine kinase inhibitor (pexidartinib,Turalia), a non-ATP-competitive “STAMP” inhibitor of the BCR-Abl fusion protein (asciminib, Scemblix), a covalently-acting G12C mutant KRAS inhibitor (sotorasib, Lumakras or Lumykras), an EZH2 methyltransferase inhibitor (tazemostat, Tazverik), and a transcription factor (HIF-2α) targeting agent (belzutifan, Welireg).

## Low-molecular-weight anti-neoplastic drugs approved by US FDA 2019–2023

In aggregate, 38 antineoplastic LMW-NMEs were approved by US FDA 2019–2023. Three of these newly approved drugs [umbralisib (Ukoniq), melphalan flufenamide (Pepaxto), and mobocertinib (Exkivity)] were omitted from this review, because each one was withdrawn from clinical use before the end of 2023. Lurbinectedin (Zepzelca), which alkylates guanine residues in the minor groove of DNA, was also omitted from our analyses. All 34 of the remaining antineoplastic LMW-NMEs approved by US FDA during this period target human proteins for which the PDB houses one or more atomic-level 3D structure. All PDB structures are freely available at no charge and with no limitations on data usage.

## Impact of PDB structures on anti-neoplastic drug approvals

We searched the PDB archive using corresponding reference amino acid sequences from UniProt (www.uniprot.org) to identify 3D biostructures that include all or part of the known macromolecular target for each of the 34 protein-targeting antineoplastic LMW-NMEs (Table 1). As of February 2024, the archive contained one or more target protein structures for all of these drug targets (34/34, 100%). More than 80% of the target protein structures for the 34 LMW-NMEs were deposited to the PDB at least a decade before the drug was approved for clinical use by US FDA. The median time between the first PDB deposition of each LMW-NME target protein structure and US FDA approval of the LMW-NME exceeded 17 years (Table 2). The LMW-NMEs themselves are also well represented in the PDB. For ~74% (25/34) of the LMW-NMEs, one or more PDB structures reveal at the atomic level precisely how the drug binds to the corresponding target protein (Table 2).

**Table 1:** Overview of PDB holdings for antineoplastic LMW-NMEs and their known molecular targets approved by US FDA 2019–2023.

LMW-NME protein target class	NMEs in protein target class	NMEs with protein target structures in PDB	Total unique PDB IDs for NME protein target structures (>99% Identity)	Number with protein target/NME co-complex structure(s) in PDB
Enzyme: Protein Kinases	24	24 (100%)	1388	20 (~83%)
Enzyme: IDH1	1	1 (100%)	48	1 (100%)
Enzyme: EZH2	1	1 (100%)	27	0 (0%)
Enzyme: γ-secretase	1	1 (100%)	19	1 (100%)
Nuclear Hormone Receptors: ERα, AR	2	2 (100%)	527	1 (50%)
GPCR: GnRHR	1	1 (100%)	1	0 (0%)
GPTase: G12C KRAS	2	1 (100%)	328	1 (50%)
Transcription Factor: HIF-2α	1	1 (100%)	32	1 (100%)
Karyopherin: XPO1	1	1 (100%)	8	1 (100%)
All	34	34 (100%)	2378	26 (~76%)

**Table 2:** Evidentiary summary for structure-guided drug (SGDD) discovery of anti-cancer LMW-NMEs approved by US FDA 2019–2023. (N.B: PDB holdings reported in Column 5 were assessed in March 2024).

NME Generic Name (TradeName)	Sponsor Company	Target Class	Target UniProt ID Oncology Indication	Earliest >95% Amino Acid Sequence Identical PDB ID for Target Protein/Year (Literature Citation) Number (PDB IDs containing human target protein)	US FDA Approval Year^a^ Delta (Years from 1st PDB ID public data release to US FDA Approval)	Target Protein/LMW-NME Complex PDB ID (Literature Citation) Year of PDB Public Data Release	Source of Target-NME Complex PDB ID	SGDD (Yes, Prob, Poss, Unl)
erdafitinib (Balversa)	Janssen	Protein Kinase	FGFR^a^ P11362 Bladder Cancer	1fgk/1996 [90] Number 70	2019 Delta 23	5ew8 [33] 2016 (Before Approval)	Academia	Yes
alpelisib (Piqray)	Novartis	Protein Kinase	PI3K P42336 Breast Cancer	2rd0/2007 [91] Number 103	2019 Delta 12	4jps [34] 2013 (Before Approval)	Industry	Yes
pexidartinib (Turalio)	Plexxikon→ Daichi Sankyo	Protein Kinase	CSF-1R P07333 TGCT	2i0v/2006 [66] Number 24	2019 Delta 13	4r7h [35] 2015 (Before Approval)	Academia	Yes
entrectinib (Rozlytrek)	Genentech	Protein Kinase	ALK Q9UM73 NSCLC	2xb7/2010 [92] Number 77	2019 Delta 9	5fto [36] 2016 (Before Approval)	Industry	Yes
zanubrutinib (Brukinsa)	BeiGene USA	Protein Kinase	BTK Q06187 Mantle Cell Lymphoma	1k2p/2001 [93] Number 129	2019 Delta 18	6j6m [37] 2019 (At Approval)	Industry	Yes
avapritinib (Avyakit)	Blueprint Medicines	Protein Kinase	PDGFRα P16234 GIST	5k5x/2016 [94] Number 13	2020 Delta 5	8pqh [46] 2024 (After Approval)	Academia	Poss
tucatinib (Tukysa)	Oncothyreon→ Seattle Genetics	Protein Kinase	Her2 P04626 Breast Cancer	3pp0/2011 [95] Number 49	2020 Delta 9	N/A	N/A	Poss
pemigatinib (Pemazyre)	Incyte	Protein Kinase	FGFR2 P21802 Cholangio Carcinoma	1gjo/2003 10.2210/pdb1gjo/pdb Number 52	2020 Delta 17	7wcl [47] 2022 (After Approval)	Academia	Prob
capmatinib (Tabrecta)	Incyte→ Novartis	Protein Kinase	MET P08581 NSCLC	1r0p/2003 [96] Number 116	2020 Delta 17	N/A	N/A	Poss
selpercatinib (Retevmo)	Lilly→ Loxo Oncology	Protein Kinase	RET P07949 NSCLC	2ivu/2006 [97] Number 34	2020 Delta 14	7ju6 [48] 2021 (After Approval)	Academia	Prob
ripretinib (Qinlock)	Deciphera Pharmaceuticals	Protein Kinase	KIT P10721 GIST	1pkg/2003 [98] Number 42	2020 Delta 17	6mob [38] 2019 (Before Approval)	Industry	Yes
pralsetinib (Gavreto)	Blueprint Medicines	Protein Kinase	RET P07949 NSCLC	2ivu/2006 [97] Number 34	2020 Delta 14	7ju5 [48] 2020 (At Approval)	Academia	Prob
tepotinib (Tepmetko)	Merck	Protein Kinase	MET P08581 NSCLC	1r0p/2003 [96] Number 116	2021 Delta 18	4r1v [39] 2015 (Before Approval)	Industry	Yes
trilaciclib (Cosela)	G1 Therapeutics	Protein Kinase (For Bone Marrow Protection)	CDK4-CKD6 P11802- Q00534 Breast Cancer	2w96/2009-1bi7/1998 [99, 100] Number 14–19	2021 Delta 12–23	N/A	N/A	Prob
tivozanib (Fotivda)	Kyowa Kirin→ Aveo Pharmaceuticals	Protein Kinase	VEGFR P35968 Renal Cell Carcinoma	1vr2/2000 [101] Number 54	2021 Delta 21	4ase [49] 2012 (Before Approval)	Industry	Yes
infigratinib (Truseltiq)	Novartis→ Bridge Biopharma	Protein Kinase	FGFR2 P21802 Cholangio carcinoma	1gjo/2003 10.2210/pdb1gjo/pdb Number 52	2021 Delta 18	3tt0 [40] 2011 (Before Approval)	Industry	Yes
asciminib (Scemblix)	Novartis	Protein Kinase Non-ATP-Competitive	BCR-Abl P00519 Ph+ CML	1fpu/2000 [69] Number 81	2021 Delta 21	5mo4 [41] 2017 (Before Approval)	Industry	Yes
futibatinib (Lytgobi)	Lilly	Protein Kinase	FGFR2 P21802 Cholangio carcinoma	1gjo/2003 10.2210/pdb1gjo/pdb Number 52	2022 Delta 19	6mzq [42] 2019 (Before Approval)	Academia	Yes
pirtobrutinib (Jaypirca)	Lilly	Protein Kinase	BTK Q06187 Mantle Cell Lymphoma	1k2p/2001 [93] Number 129	2023 Delta 22	8fll [43] 2023 (At Approval)	Industry	Yes
quizartinib (Vanflyta)	Ambit→ Daichi Sankyo	Protein Kinase	FLT3 P36888 AML	1rjb/2004 [102] Number 10	2023 Delta 19	4xuf [50] 2015 (Before Approval)	Academia	Prob
repotrectinib (Augtyro)	Turning Point Therapeutics→ BristolMyers Squibb	Protein Kinase	ROS1 P08922 ROS1-positive NSLC	3zbf/2013 [103] Number 4	2023 Delta 10	7vkn [44] 2021 (Before Approval)	Industry	Yes
momelotinib (Ojjaara)	GSK	Protein Kinase	Jak1 P23458 Myelofibrosis	3eyg/2009 [104] Number 50	2023 Delta 14	7nns (Unpublished, see doi.org/10.2210/pdb7nns/pdb) 2021 (Before Approval)	Academia	Prob
fruquintinib (Fruzaqla)	Takeda	Protein Kinase	VEGFR P35968 Colorectal Cancer	1vr2/1999 [101] Number 54	2023 Delta 24	N/A	N/A	Prob
capivasertib (Truqap)	AstraZeneca	Protein Kinase	AKT2 P31751 Breast Cancer	1o6k/2002 [105] Number 18	2023 Delta 21	4gv1 [45] 2013 (Before Approval)	Industry	Yes
olutasidenib (Rezlidhia)	Forma Therapeutics	Isocitrate Dehydrogenase	IDH1 O75874 AML	1t09/2004 [29] Number 48	2022 Delta 18	6u4j [54] 2020 (Before Approval)	Industry	Yes
tazemetostat (Tazverik)	Epizyme	Methyl transferase	EZH2 Q15910 Epithelioid sarcoma	4mi0/2013 [79] Number 27	2020 Delta 7	N/A	N/A	Poss
nirogacestat (Ogsiveo)	Pfizer→ Springworks Therapeutics	Multi-subunit Protease	Nicrastin subunit of γ-secretase Q92542 Desmoid Tumors	5a63/2015 [106] Number 19	2023 Delta 8	N/A	N/A	Unl
darolutamide (Nubeqa)	Orion Pharma→ Bayer	Nuclear Hormone Receptor	AR P10275 Prostate Cancer	1e3g/2001 [107] Number 92	2019 Delta 18	N/A	N/A	Prob
elacestrant (Orserdu)	Esai→ Takeda	Nuclear Hormone Receptor: Selective Estrogen Receptor Degrader	ERα P03372 Breast Cancer	1a52/1998 [108] Number 436	2023 Delta 25	7te7 [56] 2022 (Before Approval)	Academia	Prob
relugolix (Orgovyx)	Takeda	GPCR	GnRHR P30968 Prostate Cancer	7br3/2020 [57] Number 1	2020 Delta 0	N/A	N/A	Unl
sotorasib (Lumakras)	Amgen	GTPase	KRAS G12C P01116 NSCLC	1d8d/2000 [109] Number 329	2021 Delta 21	6oim [77] 2019 (Before Approval)	Industry	Yes
adagrasib (Krazati)	Mirati Therapeutics	GTPase	KRAS G12C P01116 NSCLC	1d8d/2000 [109] Number 329	2022 Delta 22	N/A	N/A	Yes
belzutifan (Welireg)	Merck	Transcription Factor	HIF-2α Q99814 VHL Disease	3f1n/2009 [83] Number 32	2021 Delta 12	7w80 [55] 2022 (After Approval)	Academia	Prob
selinexor (Xpovio)	Karyopharm Therapeutics	Karyopherin	XPO1 O14980 Multiple Myeloma	3gb8/2009 [58] Number 8	2019 Delta 20	7l5e [52] 2021 (After Approval)	Academia	Yes

^a^*FGFR* fibroblast growth factor receptor, *PI3K* phosphoinositide 3 kinase, *CSF-1R* colony stimulating factor-1 receptor, *TGCT* tenosynovial giant cell tumor, *ALK* anaplastic lymphoma kinase, *BTK* Bruton’s tyrosine kinase, *PDGFRα* platelet derived growth factor receptor α, *HER2* human epidermal growth factor receptor 2, *GIST* gastrointestinal stromal tumor, *MET* hepatocyte growth factor receptor, *NSCLC* non-small-cell lung cancer, *RET* rearranged during transfection receptor tyrosine kinase, *KIT* cluster of differentiation 117, *CDK-4* cyclin-dependent kinase-4, *CDK-6* cyclin-dependent kinase-6, *VEGFR* vascular endothelial growth factor receptor, *Ph+ CML* Philadelphia chromosome positive chronic myeloid leukemia, *AML* acute myeloid leukemia, *FLT3* feline McDonough sarcoma-like tyrosine kinase 3, *ROS1* proto-oncogene tyrosine-protein kinase ROS encoded by the ROS1 gene, *AKT2* AKT2 serine-threonine kinase, *IDH1* isocitrate dehydrogenase 1, *EZH2* enhancer of zeste homolog 2, *AR* androgen receptor, *ERα* estrogen receptor α, *GPCR* G-protein coupled receptor, *GnRHR* type 1 gonadotropin-releasing hormone receptor, *GTPase* nucleoside guanine triphosphate hydrolase, *KRAS* Kirsten rat sarcoma virus protein, *G12C* Glycine 12 to Cysteine, *HIF-2α* Hypoxia-inducible factor-2α, *VHL* Von Hippel Lindau, *XPO1* exportin 1.

The 34 LMW-NMEs target 9 distinct classes of proteins (Table 1), including protein kinases, three other enzymes [isocitrate dehydrogenase (IDH1), a methyltransferase (EZH2), and the nicrastin subunit of ***γ***-secretase], Exportin 1, two nuclear hormone receptors [estrogen receptor ***α*** (ER ***α***) and androgen receptor (AR)], a G-protein coupled receptor (GPCR:GnRHR), a GTPase (G12C KRAS), and a transcription factor (HIF-2***α***). In total (as of February 2024), we identified 2,378 “Relevant Structures” housed within the PDB, which include unique PDB IDs containing the following: (a) a reference or a mutant/variant form of the target protein; (b) a LMW-NME bound to a reference or mutant/variant form of its target protein; (c) a LMW-NME bound to a potential alternative target protein; or (d) a LMW-NME bound to a possible off-target protein. The number of Relevant Structures identified for each target or target class ranges from 1 for the GPCR:GnRHR to 1388 for the protein kinases.

Review of PDB archival holdings and the scientific literature pertaining to each NME target/LMW-NME combination summarized in Table 2 revealed that public domain 3D structure data facilitated discovery and development of all 34 LMW-NMEs in the following ways:

(i) Target Biology: Atomic-level 3D structures provide functional insights that are not always apparent from amino acid sequence (reviewed in [3, 22]). Maximizing understanding target biology can help avoid failures in costly Phase 3 clinical trials, wherein the biological biochemical activity of the target protein is inhibited yet the desired clinical benefit is not realized.
  In every case, the PDB houses one or more experimentally-determined atomic-level 3D structure of each NME target.
(ii) Target Druggability: Atomic-level 3D structures enable visualization of surface features deemed likely to bind small organic compounds for inhibition of enzymatic action or other interdiction of biochemical/biological function (reviewed in [4, 28]).
  In every case (34/34), PDB structures revealed one or more potential small-molecule binding sites, either on the surface of a target protein or within a protein-protein interface (e.g., the homodimeric IDH1 enzyme PDB ID 1t09 [29]). Target druggability is also informed by atomic-level, 3D structures housed in the PDB that reveal how small-molecule ligands bind to target proteins. For many of the 34 LMW-NME targets, the PDB houses co-crystal structures of the target bound to non-proprietary tool compounds (data not shown).
(iii) Structure-Guided Lead Optimization: Co-crystal structures of target protein-ligand complexes are widely used across the biopharmaceutical industry to guide optimization of potency (reviewed in [1]). In the most favorable cases, knowledge of co-complex structures with potential off targets can also be employed to help ensure the desired selectivity profile and reduce the likelihood of off-target toxicity. (For example, incidental inhibition of glycogen synthase kinase-3β (GSK-3β) causes hyperglycemia. The PDB archive houses more than 100 atomic-level 3D structures of human GSK-3β, many of which include bound small-molecules that interfere with substrate binding.) In the absence of experimental co-crystal structures of the target protein with compounds from the medicinal chemistry lead series, in silico docking tools can be used to help guide optimization of potency and selectivity (reviewed in [30]). Since the advent of the advent of the “Resolution Revolution” [31] in cryo-electron microscopy, 3DEM structures are increasingly being used as a source of information for SGDD. For cases in which an experimentally-determined 3D structure of the target protein is not available, computed structure models [14] can be combined with these same in silico docking tools. Machine learning approaches are also being used with increasing frequency to drive medicinal chemistry campaigns (reviewed in [32]).

In 28/34 ( ~ 82%) of cases, there is either direct or indirect evidence from the PDB archive (e.g., co-crystal structures), the scientific literature (e.g., mention of use of 3D structures and computational docking methods in publications), and/or private communications with industry experts to the effect that structure-guided lead optimization with the target protein reliant on experimental and/or computational tools with public domain PDB structures were used by the sponsor biopharmaceutical company or its predecessor when prosecuting the NME target (Table 2).

While it impossible to ascertain with certainty that structural data previously present in the PDB were used by the drug discovery project team, we think it to be true for every one of the new small-molecule anti-cancer agents approved by US FDA 2019–2203. In private communications with industry structural biologists, one of us (S.K.B., Director of the RCSB Protein Data Bank) has been appreciatively told on many occasions that every new drug discovery project begins with a review of relevant structures housed in the PDB. Given the sophistication of biopharmaceutical company researchers, it seems highly unlikely that public domain information with direct bearing on the task at hand would be willfully ignored when speed is of the essence and success is vital to the future of the organization.

In 25/34 (~74%) of cases, the PDB archive contains a co-complex structure of the NME bound to its target protein (Table 2), with13 coming from structural biologists based in industry and 12 coming from those based in academia (Table 2).

Not surprisingly, most of the 28 LMW-NMEs identified as confirmed or probable products of SGDD correspond to the LMW-NMEs targeting one or more protein kinase (Table 2).

Fourteen of the protein kinase inhibitors were confirmed as products of SGDD (“Yes” in Table 2) on the basis of direct evidence from the scientific literature (or private communications with industry experts) that the sponsor company or its predecessor (for acquired programs) or a competitor company used experimental and/or computational methods to understand and/or optimize how each LMW-NME bound to its target protein [33–45].

Seven protein kinase inhibitors were identified as probable products of SGDD (“Prob” in Table 2) on the basis of indirect evidence, including (a) PDB housed a structure of the target protein 10 or more years prior to drug approval; and/or (b) structural biologists based in either academia or industry deposited a co-complex structure of the LMW-NME bound to its target protein to the PDB; and/or (c) the target had been prosecuted successfully using SGDD previously by another company. We classified these less clear-cut cases as probable because we think it highly likely that the sponsor company was in possession of the same or similar data given the ubiquity of expert structural biology and computational chemistry teams across the biopharmaceutical industry today [33, 46–50].

Three kinase inhibitors were identified as possible products of SGDD (“Poss” in Table 2), because of a paucity of information. Notwithstanding lack of evidence confirming use of experimental and/or computational structural biology tools to discover these three kinase inhibitors, we think it more likely than not that SGDD played at least supporting roles during medicinal chemistry optimization of each compound. As of February 2024, the PDB housed at least 5440 structures of protein kinases, including 4817 proteins of human origin. Moreover, many sponsor companies are highly experienced in using SGDD to accelerate discovery and development of potent, selective protein kinase inhibitors. For example, nilotinib (Tasigna), the second ATP-competitive BCR-Abl inhibitor to be approved by US FDA, was the product of a SGDD campaign at Novartis [51]. (N.B.: Nilotinib’s predecessor imatinib, the first kinase inhibitor to be approved by US FDA (in 2001), was not a product of SGDD.)

The remaining seven LMW-NMEs identified as confirmed (“Yes”) or probable (“Prob”) products of SGDD target other classes of proteins (Table 2).

Three LMW-NMEs, including two GTPase inhibitors (sotorasib, Lumakras or Lumykras; adagrasib, Krazati), a nuclear export protein inhibitor (selinexor, Xpovio), and an isocitrate dehydrogenase inhibitor (olutasidinib, Rezlidhia), were confirmed as products of structure-guided drug discovery (“Yes” in Table 2) on the basis of direct evidence from the scientific literature (or private communications with experts in academia or industry) that the sponsor company used structural biology tools to study how medicinal chemistry compounds and the LMW-NME bound to its target protein [52–54].

Three LMW-NMEs, including an anti-androgen (darolutamide, Nubeqa), a transcription factor-targeting agent (belzutifan, Welireg), and an anti-estrogen (elacestrant, Orserdu), were identified as probable products of SGDD (“Prob” in Table 2) on the basis of indirect evidence [55, 56]. The evidentiary record for these drugs met identical criteria to those used above to identify the seven protein kinase inhibitors enumerated in Table 2 as probable products of SGDD.

Finally, two LMW-NMEs, nirogacestat (Ogsiveo) and relugolix (Orgovyx), were identified as unlikely to be products of SGDD (“Unl” in Table 2). For nirogacestat, a γ-secretase inhibitor, a single 3.4Å resolution 3DEM structure (PDB ID 5a63) was released only eight years prior to US FDA approval. (N.B.: There is no PDB structure containing is small-molecule.) Relugolix targets a GPCR (Gonadotropin-Releasing Hormone Receptor GNRH1R). The only human GNRH1R structure housed in the archive (PDB ID 7br3 [57]) was released in 2020 more or less coincident with NME approval. It revealed how elagolix, a compound that is structurally-similar to relugolix, binds to the same GPCR target.

The breadth and depth of PDB structures and publications coming from academia and industry revealed by our analyses reaffirms that 3D biostructure information impacts discovery of LMW-NMEs in real time. Conservative estimates suggest that MX structures of drug target proteins held as trade secrets inside company firewalls across the biopharmaceutical industry are comparable in total number to PDB archival holdings (i.e., ~220,000 structures in spring 2024). Willingness on the part of industry to share a subset of these data with academic researchers is essential for the long-term health of the experimental and computational chemistry eco-systems supporting SGDD. It is encouraging that >50% (13/25) of the PDB structures of the antineoplastic LMW-NMEs bound to their targets enumerated in Table 2 were deposited by industrial structural biology teams.

Given the highly competitive nature of biopharmaceutical industry, PDB deposition of 3D biostructures determined inside biopharmaceutical companies often lags the actual research and may not be permitted by internal policies until after drug discovery and development campaigns have succeeded. Findings summarized in Table 2 document that co-complex structures for 17 the LMW-NMEs bound to their target proteins were released into the PDB one or more years prior to US FDA approval, whereas three were released during the year of new drug approval and five were not released until at least one year following approval.

## Case studies

Going beyond these aggregate analyses, we present in brief six case studies illustrating the impact of structural biology and PDB data on discovery and development of six first-in-class antineoplastic LMW-NMEs approved by US FDA 2019–2023.

### Selinexor blockade of the exportin 1 cargo-binding groove

**Fig. 1:**
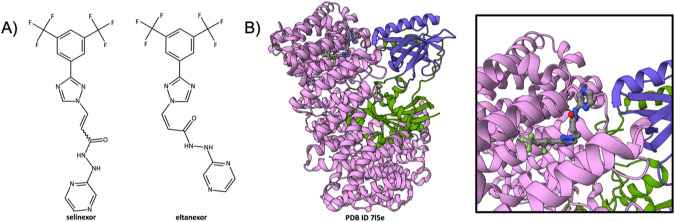
Selinexor blockade of the cargo-binding groove of XPO1. **A** Chemical structure of selinexor (left) and eltanexor or KPT-8602 (right). **B** Ribbon representation of the co-crystal structure of selinexor (Atom type color coding: C-gray; N-blue; O-red; S-yellow; F-green; Cl-green) bound to the XPO1 (pink), Ran GTP (purple), and Ran BP1 (green) heterotrimer (PDB ID 7l5e [52]). Inset Zoomed in view of selinexor bound to the XPO1 cargo binding groove. All 3D figures were generated using the Mol* Viewer [110]. Dashed line ribbons denote parts of the polypeptide chain that could not be visualized using MX. All chemical structures were drawn using the RCSB PDB Chemical Sketch Tool available at www.rcsb.org/chemical-sketch [14]. All ribbon representation drawings were prepared using the Mol* Viewer [110].

Exportin 1 (XPO1), also known as Chromosomal Maintenance 1 protein (CRM1), is a key nuclear export protein responsible for nucleocytoplasmic transport of numerous proteins and RNAs. XPO1 was identified as a promising therapeutic target in various cancers (e.g., multiple myeloma) on the strength of its role in nuclear export of tumor suppressor proteins. The first MX structure of near-full-length human XPO1 was determined at 2.9Å resolution in 2009 (PDB ID 3gb8 [58]). This structure also included the Nuclear Export Signal (NES) of snurportin, enabling significant advances in our understanding of XPO1 function. Further atomic-level insights into the function of XPO1 were provided by five related MX structures of a heterotrimeric complex of *S. cerevisiae* XPO1, human Ran, and S. cerevisiae RanBP1 bound to various cargo protein oligopeptides ranging in length from 20 to 22 amino acid residues (PDB IDs 5dhf, 5dif, 5di9, 5dha, 5dh9 [59]). Selinexor (Xpovio, Fig. 1A left), a first-in-class, orally-bioavailable covalent inhibitor of XPO1 was discovered and developed by Karyopharm Therapeutics Inc. (hereafter Karyopharm). It interferes with nuclear export of cargo proteins by occupying the cargo binding groove. Aberrant accumulation of proteins within the nucleus induces apoptosis of certain malignant cells. Karyopharm utilized structure-guided approaches to discover and develop selinexor. Karyopharm’s initial discovery efforts commenced shortly before publication of the first XPO1 structures in 2009 [58, 60]. Following public release of PDB ID 3gb8 [58], the sponsor company began using the MX structure for computational docking of small molecules. Binding of some of the compounds from the resulting lead series was visualized experimentally by Chook and co-workers, including KPT-185/PDB ID 4gmx [61], KPT-251/PDB ID 4gpt [62], and KPT-276/PDB ID 4wvf [63]. Importantly, these structures revealed a common mechanism of action (MOA) for members of the lead series distinct from that of the natural product Leptomycin B (PDB ID 4hat [64]), which failed in early stage clinical trials due toxicity concerns in the 1990s. In 2019, selinexor was granted Accelerated Approval from US FDA for use in combination with dexamethasone for treatment of adults with relapsed refractory multiple myeloma who have received at least four prior therapies, etc. The precise mechanism of action (MOA) of the drug was not revealed in 3D at the atomic level until the 2021 publication of a 1.9Å resolution co-crystal structure of selinexor (previously known as KPT-330) bound to a heterotrimeric complex of an engineered form of *S. cerevisiae* XPO1 (with a humanized cargo binding groove), human RAN, and S. cerevisiae RAN GPTase activating protein 1 (XPO1-RAN-RANBP1, PDB ID 7l5e [52], Fig. 1B and 1B Inset). At the time of writing, selinexor had been approved for use in two additional oncologic indications, and a related compound (eltanexor, Fig. 1A right, PDB ID 5jlj [65]) was under investigation by Karyopharm in clinical trial NCT02649790 (Study of the Safety, Tolerability and Efficacy of KPT-8602 in Patients With Relapsed/refractory Cancer Indications).

### Pexidartinib inhibition of CSF-1R

**Fig. 2:**
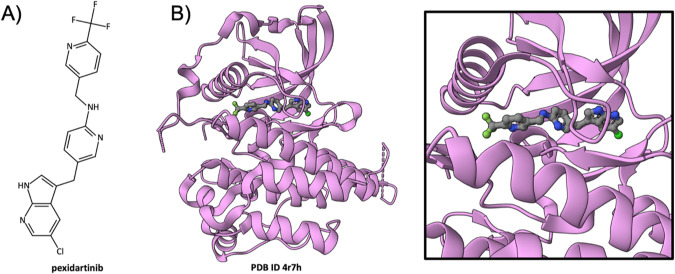
Pexidartinib inhibition of CSF-1R. **A** Chemical structure of pexidartinib. **B** Ribbon representation of the CSF-1R (pink)/pexidartinib co-crystal structure (PDB ID 4r7h [35]). Inset Zoomed in view of pexidartinib occupying the active site of the kinase catalytic domain. (Drug atom color coding and dashed line ribbons as in Fig. 1).

The Colony-Stimulating Factor-1 Receptor (CSF-1R) tyrosine kinase plays a crucial role in regulating macrophage and osteoclast production and has been implicated in tenosynovial giant cell tumor (TGCT) a rare, locally aggressive neoplasm of the joint or tendon sheath. The earliest atomic-level 3D structure of the CSF-1R kinase catalytic domain, determined at 2.8Å resolution and contributed to the PDB by industry structural biologists, was made publicly available in 2006 (PDB ID 2i0v [66]). This pioneer structure provided valuable insights into target druggability and tool compound binding (i.e., co-complex with a non-proprietary quinolone inhibitor). To target CSF-1R, Plexxikon, a pioneer in SGDD for protein kinases [67], used their approach to discover and develop pexidartinib (Fig. 2A). The drug is an ATP-competitive inhibitor of the protein tyrosine kinase activity of CSF-1R that disrupts proliferative signals contributing to uncontrolled growth of malignant cells in TGCT. A 2.8Å resolution crystal structure of the CSF-1R/pexidartinib co-complex (PDB ID 4r7h [35], Fig. 2B and 2B Inset), contributed by an academic research group, explained its MOA in 3D at the atomic level. Like many protein kinase inhibitors licensed for clinical use in oncology and other therapeutic areas, pexidartinib binds within the hinge region between the N- and C-terminal sub-domains of the kinase catalytic domain (Fig. 2B and 2B Inset), where it blocks entry of the ATP substrate (reviewed in [68]). US FDA approval of pexidartinib in 2019 marked a landmark in therapeutic management of TGCT, offering a non-surgical treatment option that could significantly improve patient outcomes.

### Asciminib Inhibition of BCR-Abl

**Fig. 3:**
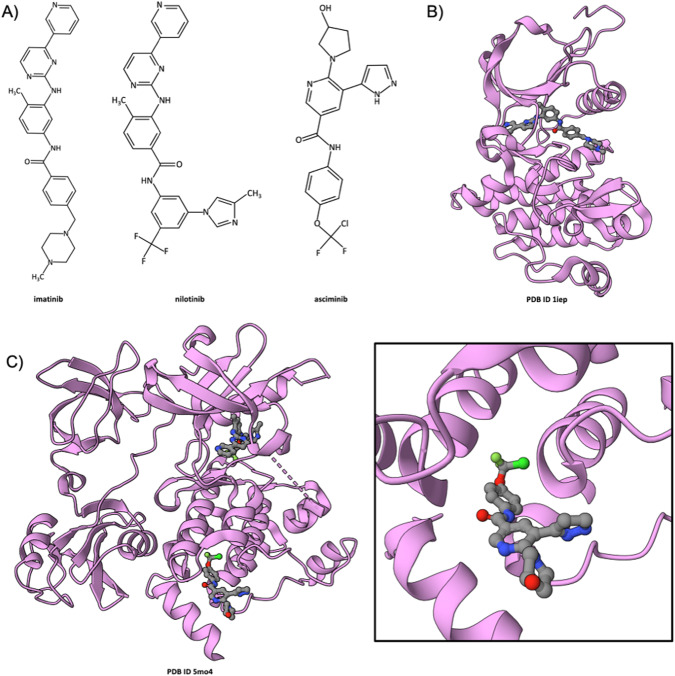
Asciminib inhibition of BCR-Abl. **A** Chemical structures of imatinib (left), nilotinib (center), and asciminib (right). **B** Ribbon drawing of the co-crystal structure of imatinib bound to murine Abl (pink) (PDB ID 1iep [70]). **C** Ribbon drawing of the co-crystal structure of human Abl (pink) bound to both nilotinib and asciminib (PDB ID 5mo4 [41]). Inset Zoomed in view of asciminib occupying the myristoyl-binding site within the kinase catalytic domain. (Drug atom color coding and dashed line ribbons as in Fig. 1).

The BCR-Abl fusion protein is the product of a balanced chromosomal translocation involving chromosomes 9 and 22, generating the so-called Philadelphia chromosome (Ph) plus the T(9;22) translocation. The resulting BCR-Abl fusion protein (BCR denoting breakpoint cluster region) is a constitutively active non-receptor protein tyrosine kinase that is the cause of Philadelphia chromosome–positive chronic myeloid leukemia (Ph+ CML). The first atomic-level MX structure of the Abl kinase catalytic domain (bound to a variant of imatinib, an ATP-competitive inhibitor) was determined in 2000 and made public by Kuriyan and co-workers (PDB ID 1fpu [69]). The same academic group determined the co-crystal structure of imatinib itself (Gleevec, an ATP-competitive inhibitor, Fig. 3A left) bound to Abl (PDB ID 1iep [70], Fig. 3B) shortly before Novartis obtained accelerated approval of the drug by US FDA. Novartis subsequently used Abl kinase domain co-crystal structures and computational chemistry tools to discover a second-generation BCR-ABL inhibitor nilotinib (or Tasigna, Fig. 3A center). Later, SGDD was also used by Novartis to discover and develop a mechanistically distinct BCR-Abl inhibitor that does not target the enzyme active site. Instead, their LMW-NME asciminib (Fig. 3A right, approved by US FDA for treatment of Ph+ CML in 2021) blocks the myristoyl-binding site within the Abl kinase domain, thereby reducing enzyme activity [71]. This approach yielded a new treatment option for patients who have developed resistance to ATP-competitive BCR-Abl inhibitors or were unable to tolerate the side effects of such agents (e.g., commonly reported imatinib side effects include edema, nausea, vomiting, muscle cramps, musculoskeletal pain, diarrhea, rash, fatigue, and abdominal pain). A 2.2Å resolution co-crystal structure of asciminib bound to the Abl kinase domain (Fig. 3C, PDB ID 5mo4 [41], contributed to PDB by structural biologists at the Genome Institute Novartis in 2016) revealed the MOA of the drug in 3 C Inset at the atomic level. This structure also explains the MOA of nilotinib, which is ATP-competitive. Asciminib has been described as a “STAMP inhibitor” (specifically targeting the Abl myristoyl pocket) that reduces enzyme activity by binding to an allosteric pocket within the kinase catalytic domain. Unlike Abl protein, which has an N-terminal myristoyl group, the BCR-Abl fusion protein encoded by the Philadelphia chromosome lacks this post-translational modification (PTM) and is not autoinhibited. A 3.4Å resolution structure of full-length Abl (PDB ID 1opl [72]), determined in 2003 revealed in atomic detail how the N-terminal myristoyl group binds to an allosteric pocket in the kinase catalytic domain. This effect stabilizes the tertiary structure of the enzyme, such that the SH2 domain (occurring near the N-terminus of the polypeptide chain) and the SH3 domain interact with the C-terminal portion kinase catalytic domain, thereby autoinhibiting enzyme activity. Asciminib received Accelerated Approval from US FDA in 2022 for treatment of Ph+ CML in chronic phase, previously treated with two or more tyrosine kinase inhibitors.

### Sotorasib inhibition of G12C KRAS

**Fig. 4:**
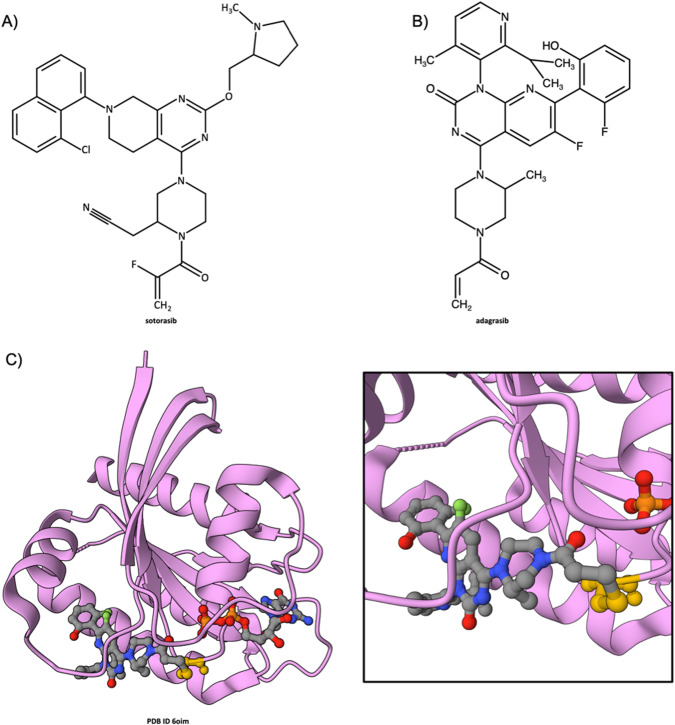
Sotorasib inhibition of G12C KRAS. **A** Chemical structure of sotorasib. **B** Chemical structure of adagrasib. **C** Ribbon representation of the co-crystal structure of sotorasib covalently bound to the G12C KRAS (pink)/GDP complex (PDB ID 6oim [77]). Inset Zoomed in view of the sotorasib binding site, showing the covalent bond (half green/half yellow) between the drug and Cysteine 12 (yellow atomic ball-and-stick figure). (Drug and GDP atom color coding and dashed line ribbons as in Fig. 1).

KRAS is one of the most frequently mutated oncogenes in human cancers, playing a critical role in regulating cell growth, differentiation, and apoptosis. Missense mutations in KRAS lead to uncontrolled cellular proliferation and tumorigenesis. Such mutations are found in ~15% of all human cancers, highlighting the pivotal role of KRAS in oncogenesis [73]. Among the many distinct KRAS mutations that have detected during tumor DNA sequencing, the Glycine12→Cysteine (G12C) change is particularly noteworthy. This somatic mutation constitutively activates KRAS and promotes uncontrolled cell growth. G12C KRAS is locked in an active conformation that drives oncogenesis. It is found in about ~30% of non-small cell lung cancer (NSLC), >85% of pancreatic cancer, and ~40% of colorectal cancer [73, 74]. The first MX structure of a closely-related HRAS protein (PDB ID 5p21 [75], a rat HRAS structure highly similar to human KRAS dating from 1990) proved critical for understanding how the human RAS enzymes (HRAS, KRAS, and NRAS) function as molecular switches that cycle between an inactive GDP-bound state and an active GTP-bound state. Normally, this cycle of activation followed by inactivation is tightly regulated by guanine nucleotide exchange factors (GEFs) like Son of Sevenless (SOS, which facilitate exchange of GDP for GTP) and GTPase-activating proteins (GAPs, which promote hydrolysis of GTP to GDP), returning KRAS to its inactive state [74]. Somatic mutations disrupt enzyme regulation resulting in continuous signaling through the RAS/RAF/MAPK/ERK signaling pathway. Notwithstanding the attractiveness of KRAS as an anti-cancer drug target, it was thought by many to be “undruggable” after multiple biopharmaceutical companies tried and failed. In 2013, however, SGDD efforts led by Shokat revolutionized KRAS inhibitor discovery and development with design of compounds that bind irreversibly to a pocket below the switch II region by engaging with the acquired cysteine of G12C KRAS, proving that KRAS activity can be inhibited with small molecules [64]. Subsequently, SGDD by Amgen yielded sotorasib (Lumakras or Lumykras, Fig. 4A left), a compound that binds selectively and irreversibly to the Cys residue of G12C KRAS [76]. PDB ID 6o8m [77] revealed the MOA of sotorasib (previously known as AMG 510) in 3D at the atomic level (Fig. 4B and 4B Inset). This first-in-class LMW-NME received Accelerated Approval from US FDA in 2021 for treatment of adults with KRAS G12C-mutated locally advanced or metastatic non-small cell lung cancer, as determined by an FDA-approved test, who have received at least one prior systemic therapy. A second covalently-acting LMW-NME targeting G12C KRAS (adagrasib, Krazat; Fig. 4A right) was approved by US FDA in 2022.

### Tazemetostat Inhibition of EZH2

**Fig. 5:**
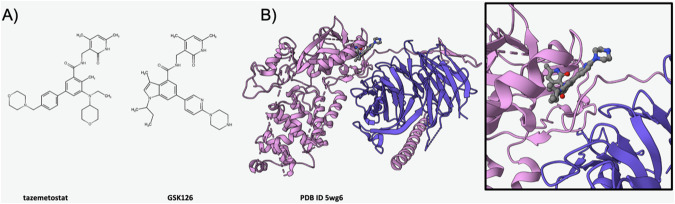
Tazemetostat inhibition of EZH2. **A** Chemical structure of tazemetostat (left) and GSK126 (right). **B** Ribbon representation of the co-crystal structure of the EZH2 (pink)/EED (purple) heterodimer bound to GSK126 (PDB ID 5wg6 [80]). Inset Zoomed in view of the SAM-binding pocket of EZH2 (PDB ID 5wg6 [80]), which is also the binding site for tazemetostat. (Drug atom color coding and dashed line ribbons as in Fig. 1).

The Enhancer of Zeste Homolog 2 (EZH2) methyltransferase is part of the Polycomb repressive complex 2 (PRC2), which includes other essential proteins such as Embryonic Ectoderm Development EED. It plays a critical role in epigenetic regulation of gene expression through methylation of Lysine 27 within the N-terminal tail of the H3 nucleosomal histone (H3K27). This PTM serves as a key signal for epigenetic gene silencing. EZH2 plays a crucial role in maintaining the balance of gene expression patterns necessary for normal cellular function. Aberrant activity of EZH2, marked by dysregulated H3K27 methylation, has been implicated in development of various cancers (reviewed [78]). In 2020, Epizyme received US FDA approval for use of tazemetostat for treatment of epithelioid sarcoma, marking a significant improvement in therapeutic options available for individuals diagnosed with this rare malignancy. The first atomic-level 3D structure of the EZH2 methyltransferase catalytic domain was released into the PDB in 2013 (ID 4mi0 [79]). Epizyme almost certainly leveraged this information during discovery and development of tazemetostat (Fig. 5A left), a first-in-class EZH2 inhibitor. Tazemetostat blocks binding of S-Adenosylmethionine (SAM, the requisite methyl donor for H3K27 modification by EZH2), reducing proliferation of malignant cells dependent on dysregulated methyltransferase catalytic activity. At the time of writing (March 2024), no public-domain structures of EZH2 with tazemetostat occupying the SAM-binding site were available from the PDB. Given public availability of atomic-level 3D structures of EZH2 seven years prior to US FDA approval of tazemetostat, we think it possible that Epizyme used SGDD during their medicinal chemistry campaign. A co-crystal structure of GSK126 (Fig. 5A right) bound to the EZH2/EED heterodimer, made public in 2018, provides detailed insights into the likely MOA of tazemetostat at the atomic level (Fig. 5B and 5B Inset, PDB ID 5wg6 [80]).

### Belzutifan inhibition of HIF-2*α*

**Fig. 6:**
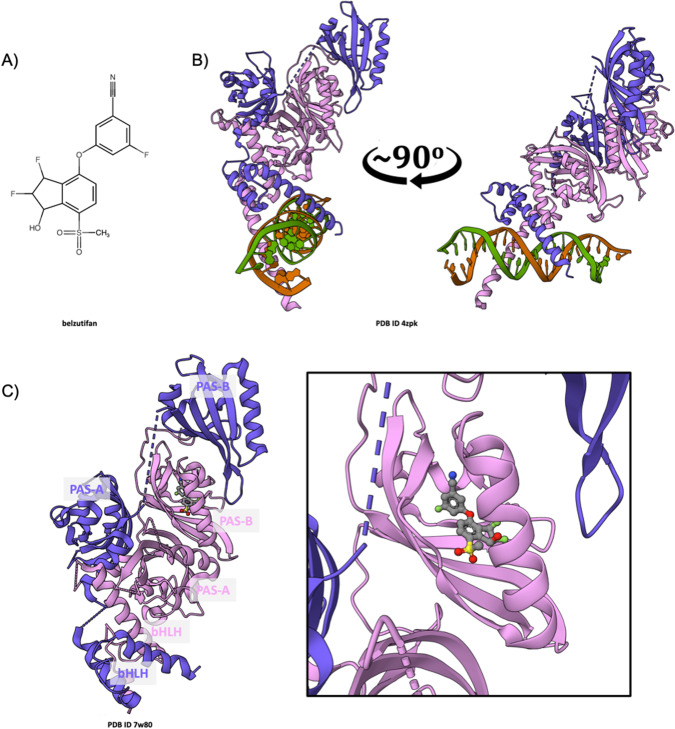
Belzutifan binding to HIF-2α. **A** Chemical structure of belzutifan. **B** Ribbon representation of the co-crystal structure of the HIF-2α (pink)/HIF- 1β (purple) heterodimer bound to duplex DNA (green and orange strands) containing an HRE (PDB ID 4zpk [85]) viewed along the helix axis (Fig. 6B left) and rotated left by ~90^o^ degree (Fig. 6B right). **C** Ribbon representation of the co-crystal structure of the heterodimer of murine HIF-2α (pink)/HIF-1β (purple) bound to belzutifan (PDB ID 7w80 [55]). Inset Belzutifan bound to HIF-2α into the PAS-B domain pocket. (Drug atom color coding and dashed line ribbons as in Fig. 1).

Hypoxia-inducible factors (HIFs) are transcription factors that regulate cellular responses to reduced oxygen availability. Among them, HIF-2*α* plays a significant role in various physiological processes and pathogenesis of certain cancers. It is the protein target of Merck’s belzutifan (Fig. 6A), which received US FDA approval in 2021 for treatment of adult patients with Von Hippel-Lindau (VHL) disease [81, 82], who require therapy for associated renal cell carcinoma, central nervous system hemangioblastomas, or pancreatic neuroendocrine tumors, not requiring immediate surgery. The first public-domain atomic-level 3D structure of human HIF-2*α* was released into the PDB in 2009 (ID 3f1n [83]) more than a decade before regulatory approval. Since then, 32 MX structures of human HIF-2*α* and 8 structures of the closely related murine HIF-2*α* have been contributed to the PDB. The mechanism by which HIF-2*α*, in a heterodimeric partnership with HIF-1β (also known as ARNT), modulates gene expression is closely tied to cellular oxygen levels. Under conditions of normoxia (normal oxygen levels), HIF-2α is hydroxylated by prolyl hydroxylase domain (PHD) enzymes. This PTM marks HIF-2α for recognition and ubiquitination by the VHL protein, leading to proteasomal degradation. Under conditions of hypoxia (reduced oxygen levels), down regulated PHD enzymatic activity results in HIF-2α stabilization and nuclear translocation. Within the nucleus it assembles into an obligate heterodimer with HIF-1β. The resulting complex engages Hypoxia-Responsive Elements (HREs) within DNA promoter regions of certain genes, initiating their transcriptional activation in response to reduced oxygen availability. The products of these genes mediate critical adaptive responses to hypoxia, including angiogenesis (*via* Vascular Endothelial Growth Factor or VEGF), erythropoiesis (*via* Erythropoietin), and metabolic reprogramming [84]. Both HIF-2α and HIF-1β are multi-domain proteins, each consisting of an N-terminal basic Helix-Loop-Helix (bHLH) segment (responsible for DNA binding), followed by two Per-Arnt-Sim or PAS domains (PAS-A, PAS-B). HRE recognition by the HIF-2α/HIF-1β heterodimer was revealed in 3D at the atomic level in a 3.6Å resolution co-crystal structure of the murine proteins bound to duplex DNA bearing an HRE (PDB ID 4zpk [85]), made public in 2015. The basic regions of each bHLH segment engage the major groove on opposite faces of the double helix (Fig. 6B), utilizing intermolecular interactions between amino acid sidechains and nucleotide base edges for HRE recognition. Targeting of HIF-2α within the heterodimer by belzutifan abrogates DNA binding, thereby disrupting signaling pathways that would otherwise be exploited by malignant cells to support growth under hypoxic conditions. The MOA of belzutifan was revealed in 3D at the atomic level in a 2.75Å resolution co-crystal structure of the drug bound to murine HIF-2α/HIF-1β (PDB ID 7w80 [55], Fig. 6C and 6C Inset) contributed by an academic research group in 2021. The NME binds in a groove on the surface of the PAS-B domain of HIF-2α (Fig. 6C and 6C Inset). Disruption of interdomain interactions within the HIF-2α/HIF-1β heterodimer would appear to explain why the drug-bound form of the heterodimer is no longer able to recognize HREs within promoter DNA and turn on the hypoxia transcriptional program necessary for tumor cell survival. Merck’s approach provides a novel therapeutic strategy for VHL-disease-related cancers, wherein the HIF-2α/HIF-1β heterodimer contributes significantly to survival and proliferation of malignant cells under hypoxic conditions. While we do not have definitive evidence that Merck used SGDD to discover and develop belzutifan, we think it probable, given the longstanding productivity of Merck structural biologists, that the company was in possession of a co-crystal structure comparable to PDB ID 7w80 during their medicinal chemistry campaign. At a minimum, open access to PDB IDs 3f1n, 4zpk, 7w80, *etc*. will facilitate SGDD efforts focused on discovery and development of second-generation NMEs targeting HIF-2α.

## Conclusion

This review documents that public-domain 3D biostructure data stored in the PDB contribute broadly to oncology drug discovery/development across the biopharmaceutical industry. For the 34 LMW-NMEs approved by US FDA 2019–2023, there is evidence from the PDB, industry experts, and/or the scientific literature that discovery and development of every one of these new drugs was facilitated by open access to experimentally-determined, atomic-level 3D structures of their protein targets housed in the PDB. In >80% of cases, the LMW-NMEs were the product of biopharmaceutical company SGDD efforts, involving co-crystal structure studies and/or computational docking using experimentally-determined crystal structures, etc.

With year-on-year growth in the number of structures in the PDB approaching 10%, the impact of the resource and structure-guided approaches to drug discovery/development is destined to remain significant. Moreover, the growing number of PDB structures coming from 3DEM since the advent of the cryo-electron microscopy “Resolution Revolution” [31], promises even broader 3D structural coverage of the human proteome. Every year at the RCSB Protein Data Bank, we witness deposition of exciting new 3D structures of integral membrane proteins and other macromolecular machines, many of which are sub-optimally targeted with relatively non-specific agents or have been held to be undruggable [86].

The long-standing requirement for PDB deposition of 3D atomic coordinates and experimental data and metadata upon journal publication ensures that this valuable information is made immediately available to basic and applied researchers around the world without limitations on usage. Moreover, expert biocuration and rigorous validation of experimental data and atomic coordinates across the PDB help to ensure that the archive as a whole can be mined for new knowledge using statistical tools [87, 88] or machine learning approaches [89].

As custodian of the PDB Archive, the wwPDB partnership is committed to the FAIR Principles [10], which help ensure the broadest possible use of public domain biomedical research data. The PDB has been recognized as a Core Certified Repository by CoreTrustSeal (coretrustseal.org). In 2022, the PDB was further recognized by the Global Biodata Coalition (https://globalbiodata.org) as a Global Core Biodata Resource, of “fundamental importance to the wider biological and life sciences community and the long-term preservation of biological data.” These two international, community-based, non-governmental, non-profit organizations promote investment in sustainable, trustworthy data infrastructure. The PDB is universally regarded as a gold-standard exemplar and a vanguard in the open access data movement in the biological and biomedical sciences.
